# Pharmacological Protection of Kidney Grafts from Cold Perfusion-Induced Injury

**DOI:** 10.1155/2019/9617087

**Published:** 2019-05-14

**Authors:** Anna Krzywonos-Zawadzka, Aleksandra Franczak, Michael A. J. Moser, Agnieszka Olejnik, Grzegorz Sawicki, Iwona Bil-Lula

**Affiliations:** ^1^Department of Medical Laboratory Diagnostics, Division of Clinical Chemistry, Wroclaw Medical University, Ul. Borowska 211A, 50-556 Wroclaw, Poland; ^2^Department of Surgery, University of Saskatchewan, Saskatoon, SK, Canada; ^3^Saskatchewan Renal Transplant Program, Saskatoon, SK, Canada; ^4^Department of Pharmacology, College of Medicine, University of Saskatchewan, Saskatoon, Canada

## Abstract

One of the greatest challenges facing the field of organ transplantation is the shortage of donor organs for transplantation. Renal transplantation increases quality of life and survival of patients suffering from end-stage renal disease. Although kidney transplantation has evolved greatly over the past few decades, a not insignificant amount of injury occurs to the kidney during recovery, preservation, and implantation and leads to the loss of function and loss of years of dialysis-free living for many patients. The use of kidneys from expanded criteria donors (ECD) and donation after circulatory determination of death (DCDD) has been adopted partly in response to the shortage of donor kidneys; however these kidneys are even more susceptible to ischemic injury. It has been shown that matrix metalloproteinases (MMPs) and reactive oxygen species (ROS) are involved in mechanisms of injury to the transplant kidney. There is also some evidence that inhibition of MMP activity and/or ROS production can protect the kidney from injury. We review possible pharmacological strategies for protection of kidney graft from injury during recovery, preservation, and implantation.

## 1. Introduction and Background

Kidney transplantation increases quality of life and life expectancy in patients suffering from end-stage renal disease compared to renal dialysis. In an effort to increase the number of kidneys available for transplantation in the face of ongoing donor organ shortage, the use of kidneys from more marginal donors has been increasing. This includes kidneys from older donors and those with cardiovascular diseases, such as hypertension (expanded criteria donors (ECD)), as well as donation after circulatory determination of death (DCDD). Both ECD and DCDD kidneys are associated with poorer initial function, a higher rate of delayed graft function (DGF) and poorer function at one year after transplantation [1]. Furthermore, it has been suggested that DGF and kidney injury of any kind are associated with an increased risk of acute rejection [2].

Although kidney transplantation has evolved greatly over the past few decades, the fact remains that, in the process of removing a kidney from a donor, flushing, and cooling it, then rapidly rewarming it once its blood vessels are connected in the recipient, significant injury occurs to the kidney and leads to loss of function as well as loss of years of dialysis-free living of these patients.

Ischemia-reperfusion injury (IRI) is an inevitable relevant outcome of kidney transplantation. Kidneys from DCDD are highly sensitive to IRI, a complex pathophysiological process involving hypoxia and following reoxygenation, ionic imbalance, oxidative stress, and mitochondrial uncoupling, as well as a coagulation and endothelium activation associated with a proinflammatory immune response. The main consequences of renal IRI are kidney graft primary nonfunction and delayed graft function or chronic graft dysfunction, all of which involve a mandatory patient's return to dialysis. The incidence of delayed graft function varies greatly, ranging from 10% when using living donor kidneys to more than 50% for DCDD kidneys [3]. In addition, the DGF is one of the more frequent early complications after the deceased-donor kidney transplantation and is primarily a consequence of postischemic acute tubular necrosis caused by IRI [4].

Minimizing kidney injury would be a tremendous benefit to patients who are waiting for kidney transplant. By potentially broadening the pool of kidneys that could be acceptable for transplantation purposes, the optimization of kidney preservation and reduction of injury is of great importance in kidney transplantation.

## 2. The Three Phases of Transplantation during Which Injury Can Occur

During the process of transplantation from one person to another, the kidneys are subjected to ischemic injury, when the blood flow supply is either interrupted or severely disturbed as well as reperfusion injury become as a consequence of the blood flow reconstruction. Injury occurs first time during the procurement (warm ischemia time, WIT I and WIT II), then a second time during the preservation (cold ischemia time, CIT), and a third time at the time of reperfusion and reoxygenation.

Warm ischemic time lasts from stopping of blood flow through the donor organ until cold perfusion is commenced (WIT I). WIT I differs regarding type of donors: it lasts just a few minutes for living donors, much longer for DCDD donors and it is minimal for donation after neurological determination of death (DNDD) donor kidneys. A period of relative ischemia between withdrawal of life supporting treatments and asystole can last from a few minutes to one and a half hours or even 2 hours depending on the program and factors specific to each DCDD donor. Prolonged time of warm ischemia in DCDD leads to greater damage of transplanted kidney compared to donation after brain death (DBD) with all the consequences: poorer initial as well as long-term functions, DGF, and higher risk of rejection [12, 13]. This emphasizes the need for strategies to minimize WIT.

WIT II relates to vascular anastomosis (from removal of the organ from ice until reperfusion in recipient) [14] and this type of injury may be reduced by faster anastomosis or keeping the kidney cool during anastomosis using Ice Bag Technique [14].

Cold ischemia time also has an impact on the graft outcome after transplantation. Prolongation of CIT may predispose to DGF and lead to reduced graft survival and even patient survival [15, 16]. Unfortunately, a significant decrease in CIT might be difficult because of logistics (transport; procedure time).

Static cold storage in which the preservation solution is infused into the organ to flush out the blood and then the organ is immersed in the preservation solution for storage remains the most often used method for kidney preservation. Numerous preservation solutions have been developed to prevent the biochemical and structural changes that occur during the process of graft cooling and reperfusion. However, the amount of time that ECD or DCDD kidneys can be safely preserved in this manner remains limited, highlighting the need for novel strategies to improve kidney preservation and recovery [17].

Machine cold perfusion allows preservation solution to circulate continuously through the kidney for 4 to 48 hours (*via* the artery). Machine cold perfusion of transplant kidneys has shown benefit in terms of both early and long-term function [17] and this benefit may be even greater in more marginal kidneys from ECD and those obtained from DCDD [18]. However, kidney injury still occurs even when machine cold perfusion is used [6]. The mechanism by which machine perfusion protects the kidney from injury is unknown; however, it has been hypothesized that it may allow continual flushing of the microcirculation, prevent the accumulation of waste products, or reduce free radical formation [19].* Regardless, the 4 to 36 or more hours' period when a kidney is “on the pump” seems to be a strategic time for pharmacological interventions to help in protecting the kidney from IRI*.

## 3. The Mechanism of Kidney Ischemia-Reperfusion Injury during the Process of Transplantation

IRI is a multifactorial process that occurs when the blood supply to an organ is impaired followed by restoration of blood flow and reoxygenation. IRI has been described in vascular and cardiac surgery, trauma, and circulatory arrest with resuscitation and kidney transplantation.

**Figure 1:**
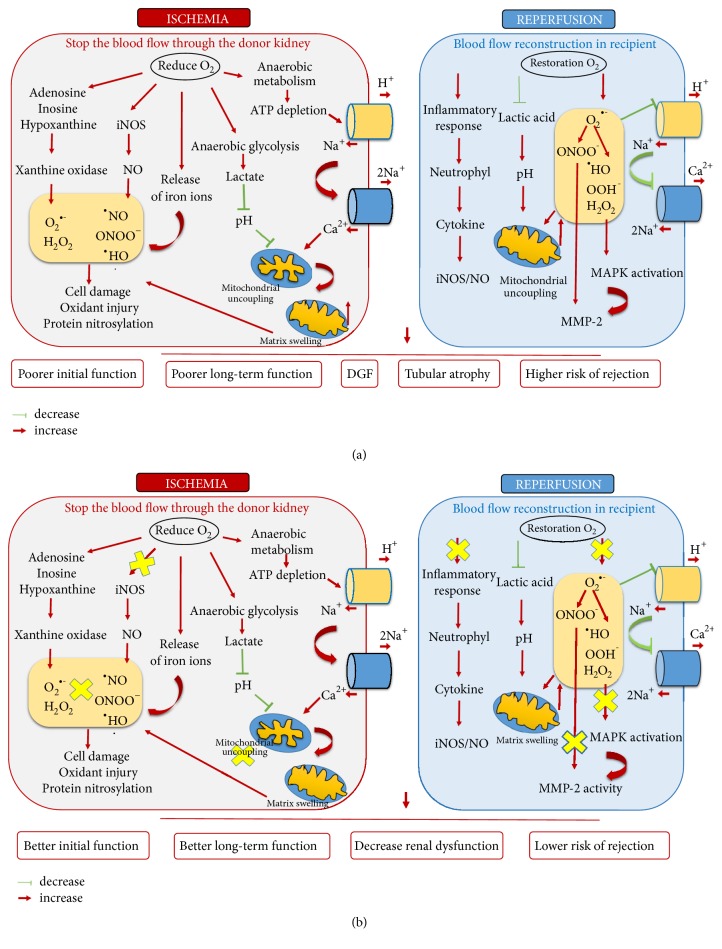
*(a) Mechanisms of renal ischemia-reperfusion injury*. The main factors contributing to kidney's damage during IRI are oxidative stress and iNOS production, a release of iron ions, accumulation of calcium in the cytosol, mitochondrial uncoupling, and inflammatory immune response. Arrows mark the increase effect (→); dash terminated with line means a decrease (*⊢*).* (b) Potential pathways of the pharmacological protection of kidney graft from ischemia-reperfusion injury*. Administration of free radical scavengers, inhibitors of iNOS and MMPs activity, or gasotransmitters are proposed to be a strategy for attenuating renal I/R injury. Yellow mark (x) indicates a potential therapy goal. DGF: delayed graft function; ATP: adenosine triphosphate; MMP-2: matrix metalloproteinase-2; O_2_^•−^: superoxide radical anion; H_2_O_2_: hydrogen peroxide; ^•^HO: hydroxyl radical; OOH: perhydroxyl radical; iNOS: inducible NO synthase; ONOO^−^: peroxynitrite; NO: nitric oxide.

The main factors contributing to kidney's damage during IRI are (a) oxidative stress, (b) accumulation of calcium in the cytosol, (c) mitochondrial uncoupling, and (d) a release of iron ions and inflammatory immune response (Figure 1).

- Ischemia leads to an increase of anaerobic metabolism, accumulation of metabolic waste products, and reduction in adenosine triphosphate (ATP) concentration, while reperfusion results in reoxygenation, and a return to aerobic metabolism. The disorders triggered during ischemia cause injury or death of tubular epithelial cells and the following reperfusion further enhances this damage as large amount of deleterious reactive oxygen species is generated [20]. ROS are toxic, highly reactive, and unstable molecules formed during a variety of normal and pathological biochemical conditions leading to DNA damage, mitochondrial malfunction, cell membrane damage, and cell death (a phenomenon referred to as “oxidative stress”).
- Prolonged ischemia increases metabolic demand and energy failure. As a consequence of anaerobic metabolism, decrease in cell pH is observed. Progressive depletion of ATP leads to inactivation of Na^+^/K^+^ ATPase and accumulation of its metabolites: adenosine, inosine, and hypoxanthine. Under nonischemic conditions, the latter one is a substrate of xanthine dehydrogenase but ionic imbalance during ischemia causes a calcium-triggered proteolytic conversion of xanthine dehydrogenase to xanthine oxidase which at the moment of reperfusion generates the superoxide free radical (O_2_^•−^) as a by-product of its action. Superoxide free radical generates secondary, more toxic reactive oxygen species: hydrogen peroxide (H_2_O_2_) and hydroxyl radical (^•^HO) [21]. Furthermore, a conformational change in endothelial cells during IRI leads to the release of iron ions which are substrates or catalysts in Fenton and Haber-Weiss reactions [22].
- Mitochondria also play an important role in the cascade of organs' damage as a result of ischemia-reperfusion. An increase of membrane permeability and thus calcium overload in the cell are observed. That triggers in turn the reduction of mitochondrial membrane potential and further inhibition of oxidative phosphorylation by impaired ATP production [23].
- Finally, ischemia-reperfusion injury is associated with neutrophil infiltration. Leukocytes mediate microvascular injury by release of proteolytic enzymes and reactive oxygen species generation. Moreover peroxynitrite, which has strong oxidizing properties, is generated during IRI. It is an additional agent triggering damage of molecules in cells including DNA and proteins [24].

All above-mentioned factors contribute to necrosis or apoptosis of tubular epithelial cells. As renal tubular atrophy occurs, progressive deterioration of kidney graft function is observed [24].

## 4. The Role of MMPs in Kidney Transplant Injury

Matrix metalloproteinases (MMPs) are a family of more than 25 structurally related proteolytic enzymes, which play important roles in a variety of physiological processes, including morphogenesis, cartilage and bone repair, wound healing, cell migration, and angiogenesis [25]. The best known MMPs are MMP-2 and MMP-9 (gelatinases) which are found in almost all cell types; they degrade and remodel collagens and other extracellular matrix proteins. Some previous studies revealed that MMPs act not only in the extracellular matrix but also at the cellular level [26].

MMPs activity is regulated at multiple levels including transcription, modulation of mRNA half-life, secretion, localization, activation, and suppression by specific and nonspecific proteinase inhibitors [26]. They are expressed as latent enzymes and can be activated by proteolytic cleavage of the N-terminal propeptide in the pericellular and extracellular compartments by a membrane-type MMPs (MT-MMP) [27, 28]. The proteolytic removal of the propeptide region perturbs the binding of a key cysteine thiol residue with the active Zn site. The disruption of this Cys-Zn^2+^ bond can be induced by ROS, such as peroxynitrite (ONOO^−^) and its derivatives. MMPs might be also regulated by naturally occurring protein inhibitors (tissue inhibitors of metalloproteinases, TIMPs) [29]. Among synthetic inhibitors of MMPs there are o-phenanthroline, hydroxamates, and tetracycline-class of antibiotics (of which doxycycline is the most potent). They all share the common characteristic of having high specificity in chelating Zn^2+^ [30]. However, they are nonselective MMPs inhibitors. As an alternative, commercially available small interfering RNA (siRNA) can be used to suppress the expression of specific genes at the mRNA level [31]. Many different siRNAs have been developed to inhibit lots of genes, also including gene for MMP-2 and MMP-9.

MMPs have long been implicated in fibrotic renal diseases. MMP-2 is involved in renal ischemia-reperfusion injury in an animal model whereby warm ischemia was induced on MMP-2 deficient transgenic mouse model [5]. The degree of acute tubular injury, necrosis, apoptosis, and renal dysfunction was markedly less in the MMP-2 deficient transgenic mice compared to that seen in the wild type mice.

MMPs play an important role in the injury of the transplanted kidney as well. Since MMPs are increased in patients with chronic antibody mediated rejection and in the fibrotic renal diseases, MMPs have been suggested to be a common pathway for chronic allograft nephropathy in the transplanted kidney [32]. On the basis of recent study showing that specific inhibition of MMPs plays a cardioprotective role during IR injury it is possible that MMPs inhibitors might have a similar protective effect on the IR damage that occurs in kidney being reoxygenated after transplantation and machine cold perfusion [33]. The use of the* ex vivo* rat model of cold perfusion confirmed that doxycycline protects the kidney from injury during cold preservation [6]. What is more, MMPs are indeed found in the perfusate of human transplant kidneys [6], supporting a possible significant role of MMPs in the injury that occurs to kidneys during cold preservation, in keeping with prior studies of fibrotic renal diseases, renal ischemia-reperfusion injury, and chronic kidney rejection (Table 1).* In conclusion, it has been shown that inhibition of MMPs may be a novel strategy for protection of the transplant kidney. The addition of pharmacological agents, as doxycycline, during preservation might reduce the damage of the kidneys [6]*.

## 5. Oxidative Stress during Renal Ischemia and Reperfusion

During ischemia, cell swelling caused by ATP depletion increases the osmotic gradient that drives water into the mitochondrial matrix to causes matrix swelling [7]. Thirty minutes of renal ischemia in rats increases matrix volume threefold and causes unfolding of cristae membranes. The loss of cristae membranes inhibits mitochondrial respiration and slows down the recovery of ATP synthesis upon reperfusion. Furthermore, electron leak in the mitochondrial electron transport chain is a major source of ROS after ischemia [7].

Oxidative stress results from an imbalance between the formation and the neutralization of prooxidants, such as superoxide radical anion (O_2_^•−^) and hydroxyl radical (HO^•^) and certain nonradicals that either are oxidizing agents or are easily converted into radicals, such as hydrogen peroxide (H_2_O_2_) and hypochlorous acid (HOCl). ROS generation triggers a cascade of reactions starting with the production of O2^•−^ which can be further converted to H_2_O_2_ by superoxide dismutases (SOD), manganese superoxide dismutase (MnSOD) in mitochondria, and copper-zinc superoxide dismutase (CuZnSOD) in cytosol. The main sinks for H_2_O_2_ are catalase (CAT) and glutathione peroxidase (GPx). The latter uses glutathione (GSH) which is oxidized to GSSG and recycled by glutathione reductase. There are other enzymes that can remove H_2_O_2_, such as peroxiredoxin/thioredoxin/thioredoxin reductase (Prx/Trx/TrxR) system. However, CAT activity is about three orders of magnitude higher compared to Prx/Trx/TrxR system which is essential under physiological settings for keeping low levels of mitochondrial H_2_O_2_ emission and for normal redox signaling* via *regulation of thiol redox switches on different proteins [34].

Increased ROS production causes activation of p38 mitogen-activated protein kinase (MAPK) signaling which participates in gene regulation of MMPs, especially MMP-2 and MMP-9 [35]. It was recently investigated that a free radical scavenger: 2-aminoethanesulfonic acid (taurine), through p38 MAPK signaling, plays a protective role in regulation of MMP-2 and MMP-9 activity in a renal I/R injury (animal model) [8].* For this reason, administration of free radical scavengers could be a strategy for attenuating renal I/R injury (regarding both oxidative stress and MMPs activity)*.

Besides ROS, reactive nitrogen species (RNS), such as nitric oxide (NO^*∙*^) and peroxynitrite (ONOO^−^), are produced in kidney IRI by inducible NO synthase (iNOS) in tubule cells. Its activity leads to producing NO in high concentration. Then, NO rapidly interacts with O_2_^•−^ to form ONOO^−^ which trigger cell damage, oxidant injury, and protein nitrosylation [36]. Numerous experimental studies have shown an increased NOS activity in kidney IRI [9]. The production of NO is important for maintaining a variety of physiological functions within the kidney as well as other solid organs. Physiologic concentrations of NO in kidney act as a tonic vasodilator but higher concentrations can be toxic, damaging cellular constituents (such as DNA) and inducing hypotension. The contributions of nitric oxide were examined in ischemia-reperfusion injury in the rat kidney. After addition of NOS inhibitors kidneys had improved renal function and reduced oxidative stress [10] (Table 1). Additionally, NOS inhibitors were documented to reduce ischemia-reperfusion injury in kidney model of DCD donor [10]. What is more, ROS significantly contribute to the rewarming injury that occurs to the transplant kidney once its blood vessels are connected in the recipient (reoxygenation).

Hence due to increased ROS/RNS production over the course of cold preservation and reoxygenation the inhibition of H_2_O_2_, ONOO^–^, and NO production might be able to protect the kidney from I/R injury during transplantation as it was shown on ischemic heart studies [37]. Studies on I/R heart model has shown that oxidative stress injury is caused by I/R, infusion of ONOO^–^ or cytokines into the myocardium increases the activity of MMP-2. This activity is positively correlated with the degree of injury. A promising new advance is the protective effect of potent scavengers of free radicals and inhibitors of lipid peroxidation administered at the time of reperfusion in a rat model of ischemic AKI. Several scavengers of ROS (e.g., superoxide dismutase, catalase, N-acetylcysteine, and edaravone) have been shown to protect against ischemic AKI in animals, but human studies have been inconclusive [36].

The relatively unsatisfactory efficiency of conventional antioxidants may be the consequence of their low penetrance to the mitochondria interior, which suffer from oxidative stress as other cellular compartments. The inner mitochondrial membrane is highly impermeable and rich in cardiolipin and maintains a strong negative internal potential that is required for the function of electron transport chain [38]. To overcome these limitations, mitochondria-targeted antioxidants have been developed to provide their delivery to the mitochondrion interior. Mitochondria-targeted antioxidants are usually chimeric molecules of a cation triphenylphosphonium (TPP) conjugated with an antioxidant moiety such as coenzyme Q10 or plastoquinone; hence, the drug concentration achieved in the mitochondrial matrix is 10,000 times higher than in the cytosol [39]. Mitochondria-targeted antioxidants have been already used in several experimental models where mitochondrial oxidative damage underlies them. One of the antioxidants, mitoquinone mesylate, has been used in phase II trials in humans regarding treatment of Parkinson disease and chronic hepatitis C showing long-term safety and tolerance [40]. Also, a targeting an antioxidant to mitochondria results in decreasing cardiac ischemia-reperfusion injury [41]. Mitoquinone mesylate, as a positively charged lipophilic cation, is accumulated in the negatively charged interior of mitochondria. Ubiquinone antioxidant component of mitoquinone mesylate is also found in coenzyme Q10. By the action of the enzyme Complex II in the mitochondrial respiratory chain, ubiquinone part of mitoquinone mesylate is rapidly activated to the active ubiquinol antioxidant. After detoxifying ROS, the ubiquinol part of mitoquinone mesylate is converted to ubiquinone, which is again subjected to Complex II to be recycled back to active antioxidant ubiquinol. This process makes mitoquinone mesylate an effective mitochondria-targeted antioxidant able to decrease heart and hepatic IRI [11]. Thus, mitoquinone mesylate added to preservation solution might prevent kidney damage during cold storage.

To effectively improve clinical outcomes of kidney transplant, gasotransmitters has recently been identified as a potential therapeutic strategy to minimize renal IRI during transplantation [42]. Gasotransmitters are the family of endogenously produced gaseous molecules that exhibits numerous antiapoptotic, antioxidant, and anti-inflammatory features that decrease IRI-associated apoptosis, oxidative stress, and inflammation, respectively. The gasotransmitters family is represented by nitric oxide, carbon monoxide, and hydrogen sulfide. Treatment of kidney donors with carbon monoxide releasing molecules (CORM-3) increases frequency of live cells and reduces cellular and graft injury through its tubular antiapoptotic effects and by upregulation of mitochondrial related Bcl-2 survival factors [43]. It has been reported that supplementation of CORM-3 at the stage of initial ischemic injury and before cold storage exposure markedly improved graft survival in models of heart and kidney transplantation. CORM-3 was identified to be protective against IRI through the reactive oxygen species upregulation and decreasing endothelial inflammation [43]. Similar effects were observed with hydrogen sulfide (H_2_S) treatment. Supplementation of standard organ preservation solution with H_2_S decreased the severity of graft injury associated with prolonged cold ischemia time in rat models of kidney transplantation. H_2_S significantly reduced the graft inflammation, expression of proinflammatory markers and progression of tissue necrosis compared to kidneys stored in standard solution. Additionally, H_2_S limits renal cellular oxidative damage through preservation of mitochondrial membrane integrity [44].* To sum up, treatment of donor kidneys with gasotransmitters (H*_*2*_*S or CORM-3) during prolonged cold storage might improve survival and recovery of allograft function during the acute posttransplant period*.

## 6. Conclusion

Renal injury during the process of transplantation is a complex issue which has become an important topic in transplantation research in recent years. The shortage of donor organs has necessitated the use of kidneys from marginal donors such as DCDD and ECD donors. However, these organs are more sensitive to injury and DCDD or ECD transplantations result in inferior outcomes compared with living donor kidneys and DNDD kidneys. Therefore, the ability to minimize of graft injury would be a tremendous benefit to patients who are waiting for a kidney transplant by helping with initial function, by potentially increasing the longevity of kidney transplants, and by broadening the donor pool of kidneys that could be acceptable for transplant purposes. In the case of ECD and DCDD donor kidneys, the optimization and reduction of WIT and CIT have become an important topic in transplantation.

Machine cold perfusion of transplant kidneys has shown benefits in terms of both early and long-term function of the organ [17]. The use of doxycycline, hydrogen sulfide, or carbon monoxide releasing molecules is showing promise in the reduction of injury and the time that a kidney is on the machine perfusion apparatus is an opportunity for pharmacological treatment and prevention of graft injury. A combination approach may lead to additive protective effects through the prevention of injury through different mechanisms.

Oxidative stress is an element of the cascade of processes participating in IRI. What is more, an excessive production of ROS and RNS not only directly participates in tissue damage but also has an influence on MMPs activity [8]. The oxidative stress leads to posttranslational modifications of different proteins which make them substrates for MMPs [45]. Many researchers have documented the role of MMPs in ischemic damage in the heart, but the basic molecular and cellular mechanisms of all IRI are similar throughout the body. The MMPs' contribution to preservation injury was confirmed in study where it was observed that the amount of MMP-2 and MMP-9 in the perfusates from human kidneys was almost double for those from DCDD donors as opposed to DBD, with minimal warm ischemia at the time of procurement [6].

Pharmacological prevention of renal IRI can result in the ability to use some of marginal donor kidneys that normally would be rejected during qualification for transplantation and help to decrease the gap between the numbers of patients on the wait list and the number of donor organs that are able to be transplanted.

## Figures and Tables

**Table 1:** Pharmacological strategies in the prevention of kidney ischemia-reperfusion.

Study	Species	Model	Ischemia (min)	Reperfusion	Inhibitor	Dose	Effect on kidney function
[5]	Knockout MMP-2^−/−^ mice	In vivo	60	24h	Minocycline	45mg/kg	↓ ATI ↓ Renal dysfunction
[5]	Knockout MMP-2^−/−^ mice	In vivo	60	24h	MMP-2/MMP-9 Inhibitor III	2.5mg/kg	↓ ATI ↓ Renal dysfunction
[6]	Rat	In vivo	10	-	Doxy	100*µ*M	↓ LDH ↓ NGAL ↓ Cco
[6]	Rat	In vivo	10	-	MMP-2 siRNA	10*µ*M	↓ LDH ↓ NGAL ↓ Cco
[7]	Rat	In vivo	45	4wk	SS-20	2mg/kg	↓ Cytoskeletal breakdown ↓ Mitochondria matrix swelling
[8]	Rat	In vivo	60	6h	Taurine	200mg/kg	↓ Degeneration tubular architecture ↓ Inflammatory cell infiltration ↓ Renal dysfunction
[9]	Mice	In Vivo	45	Up to 7 days	Everolimus	0.25mg/kg	↓ Kidney function ↓ Recovery of kidney function
[10]	Pig	In vivo	25	18h	1400W	10mg/kg	↑ Renal function ↓ Oxidative stress
[11]	Mice	In Vivo	45	Up to 24h	MitoQ	4mg/kg	↓ Oxidative damage

SS-20: H-Phe-D-Arg-Phe-Lys-NH_2_; Taurine: 2-aminoethanesulfonic acid; Everolimus: RAD001; DOXY: doxycycline; wk: weeks; ↑: increased; ↓: decreased.
